# Clinical and prognostic significance of m6A hypomethylation and *IGF2BP3* overexpression in gastric cancer: an integrated epigenomic-transcriptomic analysis

**DOI:** 10.1186/s40246-025-00802-0

**Published:** 2025-08-22

**Authors:** Xiangchen Hu, Zhe Wang, Youwei Kou, Yujing Huang

**Affiliations:** 1 Department of Radiology, Shengjing Hospital of China Medical University, Shenyang, Liaoning People’s Republic of China; 2 Department of Pathology, Shengjing Hospital of China Medical University, Shenyang, Liaoning People’s Republic of China; 3 Department of General Surgery, Shengjing Hospital of China Medical University, Shenyang, Liaoning People’s Republic of China; 4 Virology Laboratory, Shengjing Hospital of China Medical University, Shenyang, Liaoning People’s Republic of China

**Keywords:** Gastric cancer, m6A modification, RNA methylation, MeRIP-seq, IGF2BP3

## Abstract

**Background:**

Gastric cancer (GC) ranks as the fifth most prevalent malignancy and the third leading cause of cancer-related mortality worldwide, with complex pathogenesis driven by genetic and epigenetic alterations. While genetic contributors to GC have been extensively studied, the functional role of N6-methyladenosine (m6A) RNA methylation—the most abundant eukaryotic RNA modification—in gastric carcinogenesis remains insufficiently characterized. This study aimed to investigate transcriptome-wide m6A methylation dysregulation and its mechanistic implications in GC progression.

**Methods:**

Methylated RNA immunoprecipitation sequencing (MeRIP-seq) was performed to map m6A epitranscriptomes in paired GC and adjacent normal tissues. Gene Ontology (GO) and Kyoto Encyclopedia of Genes and Genomes (KEGG) pathway analyses were conducted on 253 hypomethylated mRNAs to delineate the biological pathways associated with m6A dysregulation. The transcriptomic profiles were analyzed using RNA-seq, while a retrospective cohort analysis (*n =* 58) evaluated the correlations between *IGF2BP3* expression and the clinicopathological characteristics of patients with GC.

**Results:**

MeRIP-seq analysis demonstrated transcriptome-wide m6A hypomethylation in GC tissues, identifying 271 significantly reduced peaks (*p* < 0.01). Functional annotation revealed enrichment of hypomethylated transcripts in metabolic pathways and transcriptional dysregulation. Notably, m6A-related genes exhibited widespread activation in GC, with *IGF2BP3* showing the most pronounced upregulation (106-fold increase, *p* < 0.0001). Clinically, elevated *IGF2BP3* expression significantly correlated with lymph node involvement (*p* = 0.016) and advanced TNM staging (*p* = 0.028).

**Conclusion:**

This study establishes m6A methylation dysregulation as a critical mechanism in GC pathogenesis and identifies *IGF2BP3* as both a potential therapeutic target and a prognostic biomarker in GC.

**Supplementary Information:**

The online version contains supplementary material available at 10.1186/s40246-025-00802-0.

## Introduction

N6-methyladenosine (m6A), the most prevalent mRNA modification in eukaryotic cells [1, 2], regulates various fundamental biological processes of mRNA, including decay, stability, translation, transport, and splicing [3, 4]. Despite its widespread occurrence, the functional mechanisms of m6A remain incompletely understood. Emerging evidence implicates m6A dysregulation in cancer pathogenesis, mediated by three key regulatory protein classes: “writers” (methyltransferases), “erasers” (demethylases), and “readers” (m6A-binding proteins) [5–7]. These factors dynamically modulate m6A deposition, removal, and recognition, respectively, and their aberrant expression has been linked to tumorigenesis and malignant progression [6, 8]. METTL3 and METTL14 have been identified as “writers” that mediate m6A deposition in mRNAs [9, 10]. Downregulation of METTL3 or METTL14 enhances the proliferative and renewal capacity of glioblastoma stem-like cells [11, 12], while METTL3 overexpression drives the dissemination and metastasis of lung cancer cells [13]. Studies have indicated that the progression of hepatocellular carcinoma is associated with aberrant m6A modifications, with METTL3 frequently being upregulated and contributing to disease progression [14, 15]. However, the role of m6A methylation in gastric cancer (GC)—the fifth most common malignancy and the third leading cause of cancer-related deaths globally [16, 17]—remains incompletely understood.

Although the functions of canonical m6A regulators have been partially elucidated, transcriptome-wide comparisons of m6A modifications between GC and normal tissues have not been determined to date. Recent advances in methylated RNA immunoprecipitation sequencing (MeRIP-seq) have allowed for the intensive profiling of transcriptome-wide m6A modifications [18].

In this study, we employed MeRIP-seq to map the transcriptome-wide m6A methylome in GC, identifying differentially methylated mRNA peaks relative to adjacent normal tissues. Functional annotation and pathway analyses were performed to elucidate the biological implications of m6A dysregulation. Additionally, RNA sequencing (RNA-seq) revealed differentially expressed mRNAs and associated pathways. Finally, we assessed the expression of m6A methylation regulators to determine their influence on GC progression.

## Method

### Patients and samples

Paired tumor samples and histologically normal adjacent tissues (collected ≥ 5 cm from the tumor margin) were obtained from patients with GC who underwent surgical resection at the Shengjing Hospital of China Medical University. All specimens underwent histopathological confirmation by certified pathologists. Immediately following resection, tissues were separated into sterile centrifuge tubes and flash-frozen in liquid nitrogen for preservation. This study was conducted in accordance with the Declaration of Helsinki and approved by the Review Committee of the Shengjing Hospital of China Medical University (approval number: 2022PS412K). Written informed consent was obtained from all participants after full disclosure of the study objectives and procedures.

### MeRIP-Seq

Total RNA (300 µg) was extracted from each sample and assessed for integrity (Agilent 2100 Bioanalyzer) and concentration (SimpliNano spectrophotometer, GE Healthcare). Fragmented mRNA (-100 nt) was incubated for 2 h at 4 °C with anti-m6A polyclonal antibody (Synaptic Systems) for immunoprecipitation. Then, the immunoprecipitated mRNA or inputs were used for library preparation with the NEBNext Ultra RNA Library kit (New England Biolabs) to prepare mRNA for sequencing. According to the manufacturer’s instructions, library preparations were sequenced on Illumina NovaSeq/HiSeq platforms (150 bp paired-end reads). Sequencing was performed with three independent biological replicates.

### MeRIP-qPCR

To validate the MeRIP-seq findings, MeRIP-qPCR was performed following the same protocol for RNA extraction, quality control, fragmentation, and m6A immunoprecipitation as described for MeRIP-Seq. After immunoprecipitation, the enriched m6A-modified RNA was reverse transcribed using the High-Capacity cDNA Reverse Transcription Kit (Applied Biosystems). Quantitative PCR was performed on the 7500 Fast Real-Time System (Applied Biosystems) with the following cycling conditions: 95 °C for 30 s, followed by 40 cycles of 95 °C for 10 s and 60 °C for 15 s. The relative abundance of m6A-modified transcripts was normalized to input RNA and calculated using the 2^−ΔΔCT^ method, with GAPDH served as the endogenous control. Primer sequences for the selected genes were designed using Primer-BLAST (NCBI) and are listed in Additional file 1: Table S1.

### Transcriptomic sequencing

Briefly, the mRNA was purified using poly T oligo-attached magnetic beads. First-strand cDNA was synthesized using random hexamer primers and M-MuL V Reverse Transcriptase. Following adenylation of 3’ ends of DNA fragments, adapters with hairpin loop structure were ligated to prepare for hybridization. PCR amplification was conducted using Phusion High-Fidelity DNA polymerase with universal and index primers. Purified PCR products were quality-controlled on an Agilent Bioanalyzer 2100 system. Indexed libraries were clustered on an Illumina cBot system using TruSeq PE Cluster Kit v3 and subsequently sequenced on an Illumina NovaSeq platform (150 bp paired-end).

### Sequencing data analysis

Raw sequencing reads (FASTQ format) were processed using FASTQ software (v0.19.11) to remove adapter-contaminated, poly-N, and low-quality reads. Resulting clean reads were simultaneously calculated for quality metrics (Q20, Q30 scores, and GC content).

The reference genome index was built using BWA software (v0.7.12), followed by alignment of clean reads. ExomePeak R package (v2.16.0) was used for m6A peak identification in each group, with input samples serving as controls (*p* < 0.05 significance threshold). m6A peak density was quantified as peaks per kb of transcript length and normalized by RPKM to account for sequencing depth and transcript length variations. Differential peak analysis between GC and normal tissues was performed using MACS2 (v2.1.1) (|log2FC| >1, *p* < 0.05), with batch effects corrected via the limma R package. Peaks were retained only if detected in ≥ 2/3 replicates per group to ensure reproducibility. The distribution of peaks across genomic regions (CDS, 3’UTR, etc.) was compared using chi-square tests. Functional enrichment analysis of hypomethylated genes was conducted using GOseq (v1.50.0) and KOBAS (v3.0) (corrected *p* < 0.05).

### RNA extraction and qPCR

Total RNA was isolated from paired GC and adjacent normal tissues using TRIzol reagent (Invitrogen). For each patient sample, total RNA was extracted in a single isolation procedure, aliquoted, and subsequently used for both MeRIP-seq and RNA-seq analyses. This approach ensured that all comparisons between methylation status and transcript abundance were performed using identical RNA sources, eliminating potential variability from separate isolations. Reverse transcription was performed with the High-Capacity cDNA Reverse Transcription Kit (Applied Biosystems). Quantitative PCR analysis was conducted on the 7500 Fast Real-Time System (Applied Biosystems) under the following conditions: initial denaturation at 95 °C for 30 s, followed by 40 cycles of 95 °C for 10 s and 60 °C for 15 s. Gene-specific primers were designed according to previous studies [19]. Primer sequences were: *IGF2BP1* (Forward: 5’-GACCCCTGAATGAAGAACGA-3’; Reverse: 5’-TGGTTACTCTGTCCCTTCTGA-3’); *IGF2BP2* (Forward: GGA CTT GAG CCC TGA ACC A; Reverse: TGA AAA TTC CCG TGA GAA GC); *IGF2BP3* (Forward: AGT TGT TGT CCC TCG TGA CC; Reverse: GTC CAC TTT GCA GAA CCT TC); and GAPDH (Forward: TGG TAT CGT GGA AGG ACT CA; Reverse: CCA GTA GAG GCA GGG ATG AT). Relative mRNA expression levels were calculated using the 2^−ΔΔCT^ method, with GAPDH serving as the endogenous control.

### Statistical analysis

The relative expression levels of *IGF2BP3* mRNA between GC and matched tumor-adjacent normal tissues were analyzed using paired Student’s *t*-tests. Associations between *IGF2BP3* expression and clinicopathological characteristics were evaluated by χ² tests. All statistical analyses were conducted using SPSS (v22.0; IBM), with two-tailed *p* < 0.05 considered statistically significant.

## Results

### Transcriptome-wide Reduction of m6A Methylation in GC Tissues

**Fig. 1:**
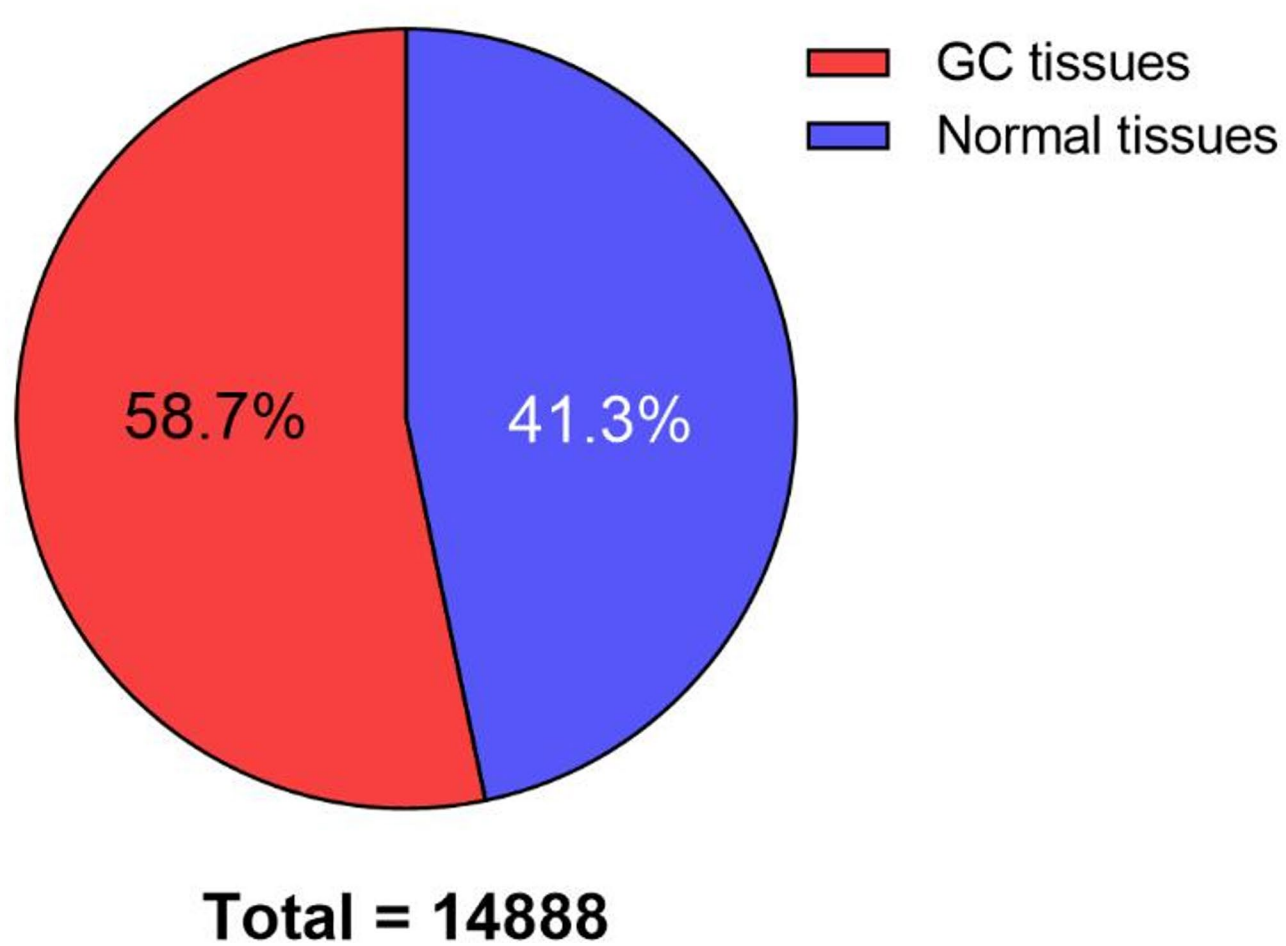
The percentage distribution of the 14,888 m6A peaks between GC and adjacent normal tissues

**Fig. 2:**
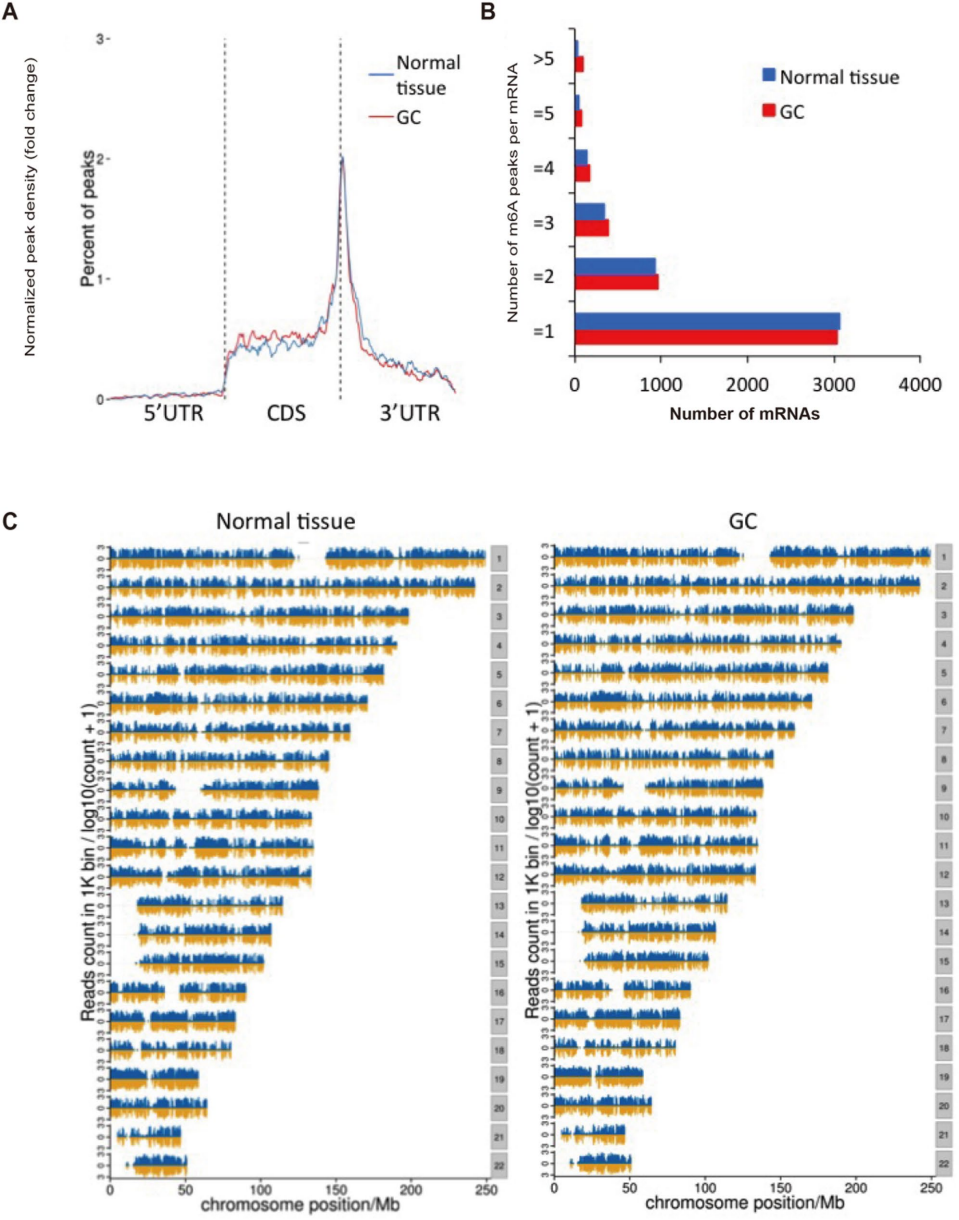
Epitranscriptomic landscape of N6-methyladenosine (m6A) peaks in GC and adjacent normal tissues. (**A**) Regional distribution of m6A peaks along the transcripts. Y-axis values (1, 2, 3): 1 = Baseline density (no enrichment); 2 = 2-fold enrichment relative to baseline; 3 = 4-fold enrichment. (**B**) Quantitative analysis of m6A peaks per mRNA transcript. **C** Chromosomal mapping profile of identified m6A peaks

**Fig. 3:**
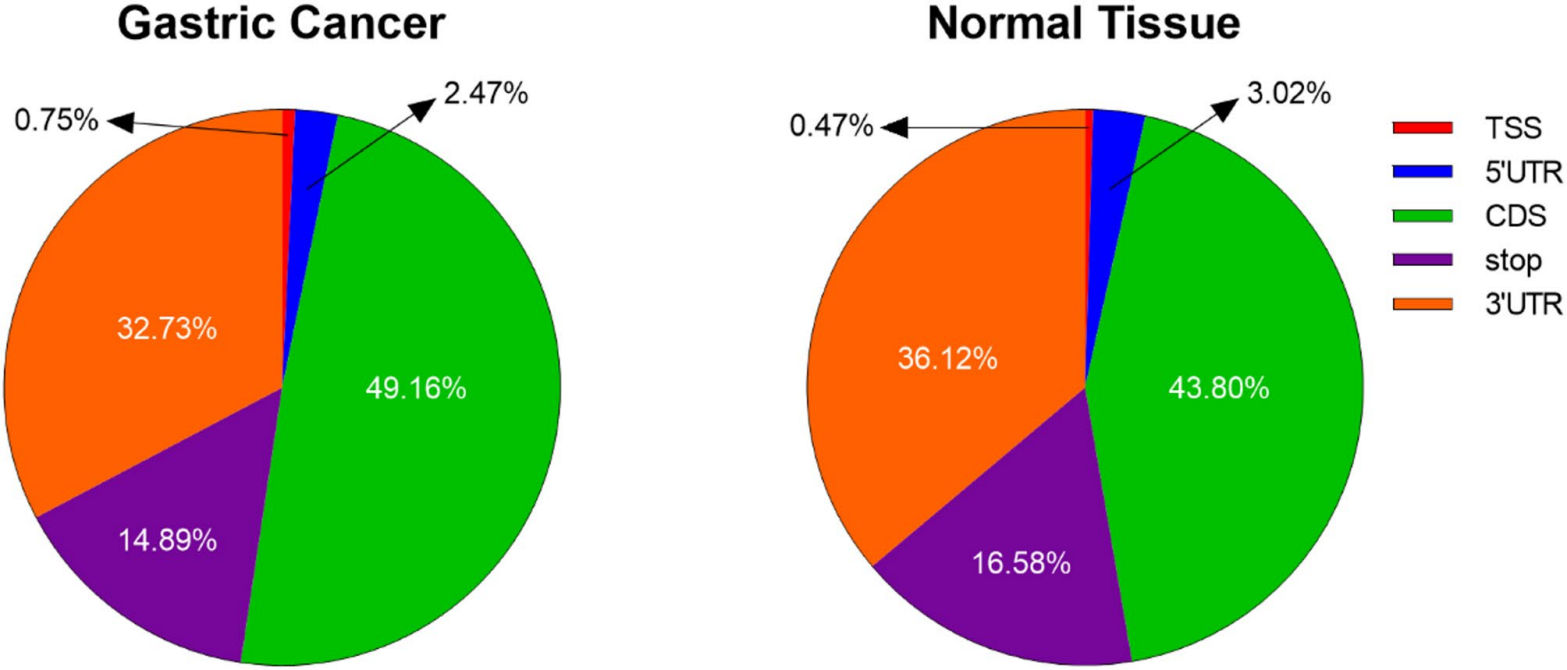
The percentage distribution of m6A peaks across different mRNA regions—TSS, 5′UTR, CDS, Stop, and 3′UTR—for both GC and normal tissues

We conducted MeRIP-seq analysis on paired tumor and adjacent normal tissues from three GC patients with GC, identifying 14,888 m6A peaks across both tissue types. The analysis revealed that 58.7% of peaks were identified in GC tissues, while 41.3% were detected in normal tissues (Fig. 1). The m6A modifications exhibited consistent transcriptome-wide distribution patterns, with predominant enrichment in coding sequences (CDS) proximal to stop codons in both tissue types (Fig. 2A). While the number of mRNAs was remarkably conserved between GC and normal tissues (Fig. 2B), quantitative analysis revealed significantly elevated m6A peak density in CDS regions of GC samples (49.16% vs. 43.80%) (Fig. 3). All m6A peaks were mapped to human chromosomes (Fig. 2C).

**Fig. 4:**
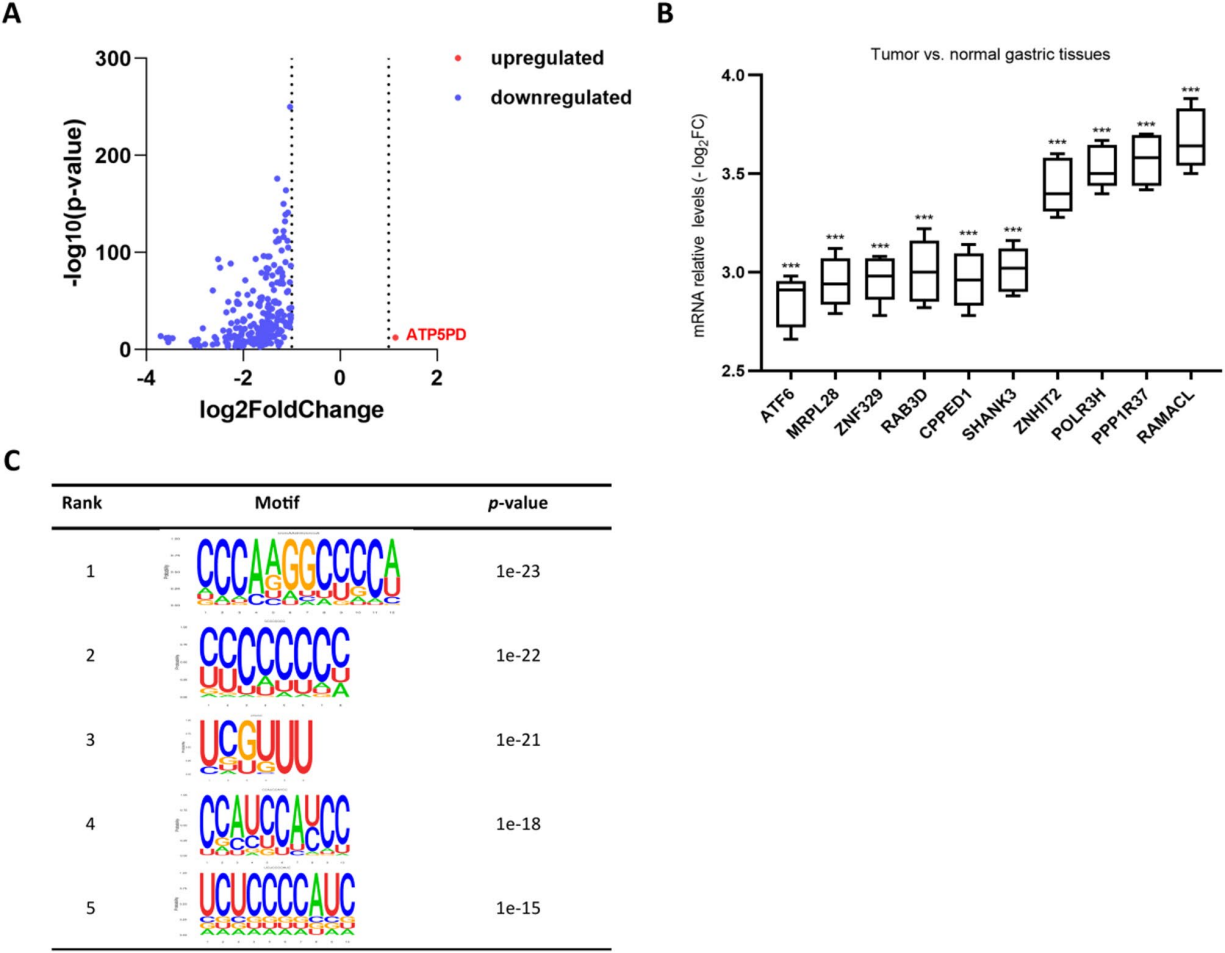
Analysis of dysregulated m6A peaks in GC. (**A**) Volcano plot analysis of significantly dysregulated m6A peaks (red: upregulated; blue: downregulated) in GC versus adjacent normal tissues. (**B**) MeRIP-qPCR validation of top regulated genes with m6A peaks in GC and normal tissues. ***, *p* < 0.0001. (**C**) Top five significantly enriched m6A consensus motifs identified from differentially methylated peaks

Volcano plot analysis (Fig. 4A) revealed identified dysregulated m6A peaks between GC and normal tissues, with only ATP synthase subunit D (*ATP5PD*) demonstrating exclusive upregulation in GC. Genome-wide profiling identified 271 significantly downregulated peaks corresponding to 253 protein-coding genes, with the most regulated genes with m6A peaks detailed in Table 1. The MeRIP-qPCR results recapitulated the MeRIP-seq findings, showing significant m6A reduction in GC tissues for all tested genes (*p* < 0.0001) (Fig. 4B). Motif enrichment analysis (Fig. 4C) delineated consensus m6A modification patterns among differentially methylated transcripts, with the top-ranked motifs exhibiting high statistical significance (*p*-values ranging from 1 × 10⁻²³ to 1 × 10⁻¹⁵).

**Table 1:** Top regulated genes with m6A peaks in GC samples

Gene Name	Chromosome	Strand	ChromStart	ChromEnd	Log2.FC	*p*-value
*ATP5PD*	chr17	-	75,038,862	75,039,247	1.14	2.42E-07
*RXRA*	chr9	+	134,439,445	134,439,656	-2.36	1.30E-08
*ARHGEF17*	chr11	+	73,309,994	73,310,354	-2.41	2.00E-20
*CBX6*	chr22	-	38,866,242	38,866,633	-2.48	3.20E-85
*ABHD17A*	chr19	-	1,876,989	1,877,289	-2.52	6.30E-94
*ZNRD2*	chr11	+	65,571,569	65,571,750	-2.54	6.20E-07
*DCAF11*	chr14	+	24,115,468	24,115,739	-2.56	1.60E-11
*MMAA*	chr4	+	145,639,203	145,639,564	-2.59	1.60E-13
*AP5Z1*	chr7	+	4,791,307	4,791,458	-2.63	7.40E-06
*RAB11FIP4*	chr17	+	31,531,104	31,531,913	-2.63	1.30E-61
*ALKBH7*	chr19	+	6,372,823	6,373,001	-2.79	3.90E-06
*ATF6*	chr1	+	161,962,754	161,962,904	-2.89	1.00E-49
*MRPL28*	chr16	-	371,079	371,289	-2.93	6.60E-06
*ZNF329*	chr19	-	58,128,015	58,129,034	-2.93	3.20E-11
*RAB3D*	chr19	-	11,322,663	11,322,843	-2.99	2.00E-09
*CPPED1*	chr16	-	12,663,903	12,664,024	-3	7.90E-05
*SHANK3*	chr22	+	50,730,994	50,731,593	-3.06	4.40E-09
*ZNHIT2*	chr11	-	65,116,432	65,116,700	-3.46	2.00E-12
*POLR3H*	chr22	-	41,533,534	41,539,260	-3.55	3.00E-08
*PPP1R37*	chr19	+	45,145,387	45,145,657	-3.59	1.30E-12
*RAMACL*	chr6	-	166,586,197	166,586,406	-3.7	1.30E-14

### The biological significance of genes with M6A downregulation in GC tissues

**Fig. 5:**
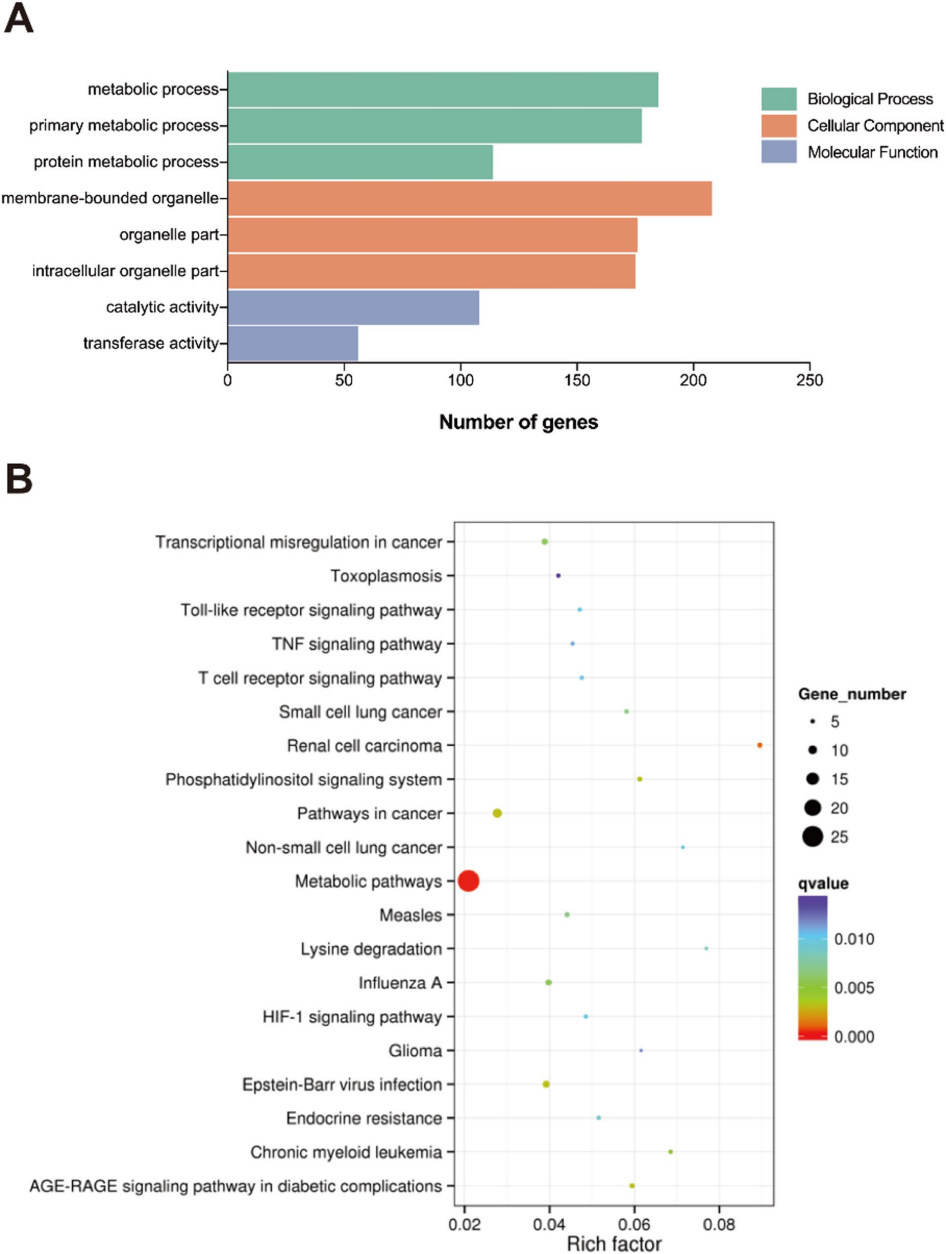
Functional enrichment analysis of m6A-hypomethylated genes in GC tissues. (**A**) GO enrichment analysis showing significant association with biological processes, cellular components, and molecular functions. (**B**) KEGG pathway analysis revealing predominant involvement in metabolic pathways, transcriptional misregulation in cancer, and cancer signaling pathways

To elucidate the biological implications of m6A hypomethylation in GC, we conducted Gene Ontology (GO) and Kyoto Encyclopedia of Genes and Genomes (KEGG) analyses on 253 hypomethylated mRNAs in GC samples compared to adjacent normal tissues. GO analysis revealed significant enrichment of these transcripts in catalytic activities (molecular function) and primary metabolic processes (biological process) (Fig. 5A). KEGG pathway analysis identified three major oncogenic networks significantly associated with m6A downregulation: metabolic pathways, transcriptional misregulation in cancer, and cancer signaling pathways (Fig. 5B).

### Different functional patterns between transcriptomes in GC tissue and the M6A methylome

**Fig. 6:**
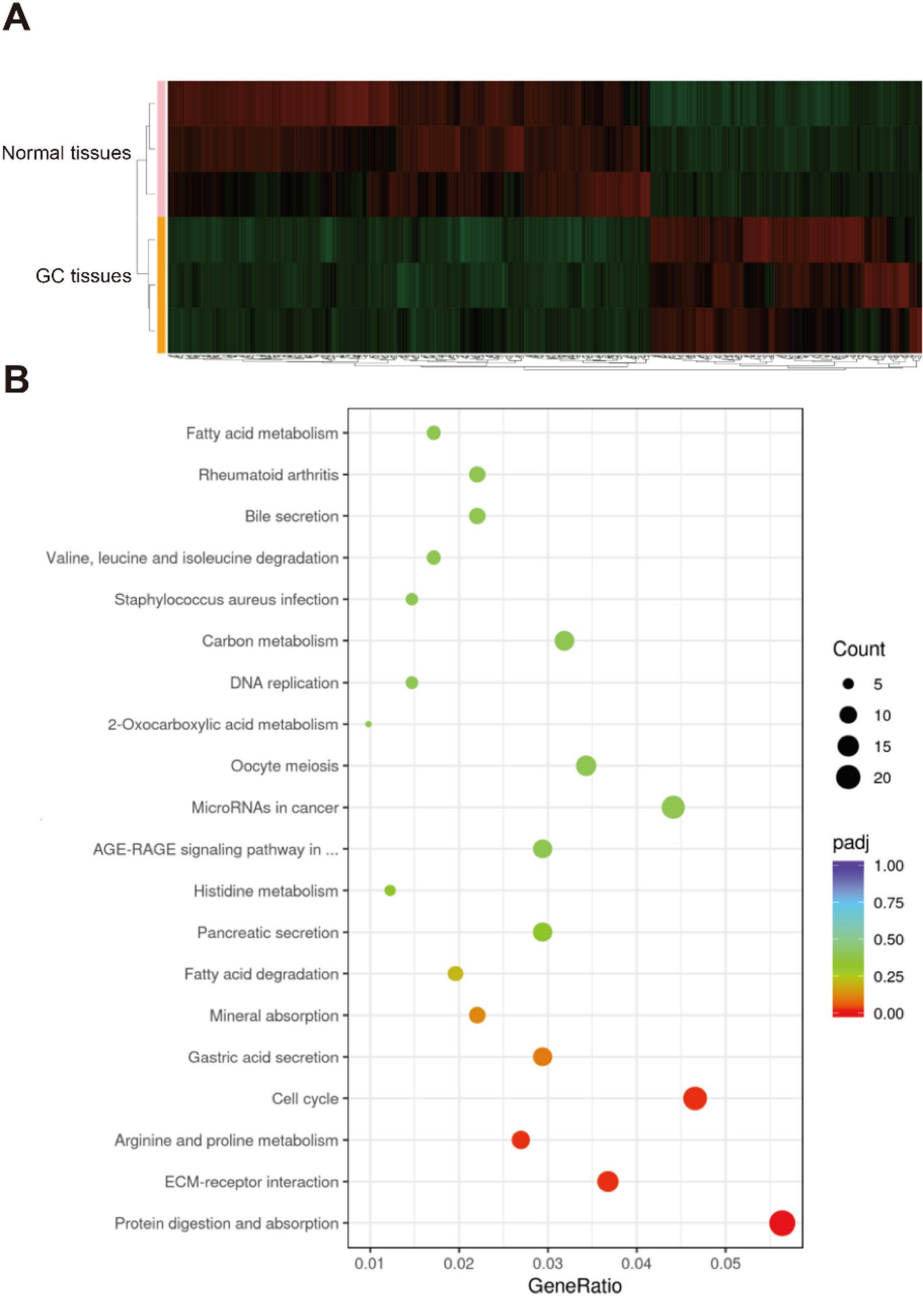
Analysis of transcriptomes in GC and normal gastric tissues. (**A**) Heatmap visualization of DEGs. (**B**) KEGG pathway enrichment analysis of mRNAs altered significantly in GC

The transcriptomic profiles of GC and adjacent normal tissues were analyzed using RNA-seq. Compared with adjacent normal tissues, 436 genes were significantly upregulated and 721 genes were downregulated in GC tissues (|log2foldchange| >0.585 and *p* < 0.05) (Fig. 6A). Integrative analysis with m6A methylome data identified five transcripts (*TOM1L2*, *ST5*, *STEAP3*, *LDOC1*, and *GLUL*) exhibiting coordinated downregulation of both mRNA expression and m6A modification levels.

Notably, KEGG pathway analysis revealed distinct enrichment patterns among differentially expressed genes (DEGs) (Fig. 6B), with significant representation in: (i) protein digestion and absorption, (ii) extracellular matrix (ECM)-receptor interactions, (iii) gastric acid secretion, and (iv) cell cycle regulation. This pathway signature markedly differs from the metabolic predominance observed in m6A-modified transcripts, suggesting independent regulatory mechanisms in gastric carcinogenesis.

### The M6A modification regulator IGF2BP3 is increased in GC samples

**Fig. 7:**
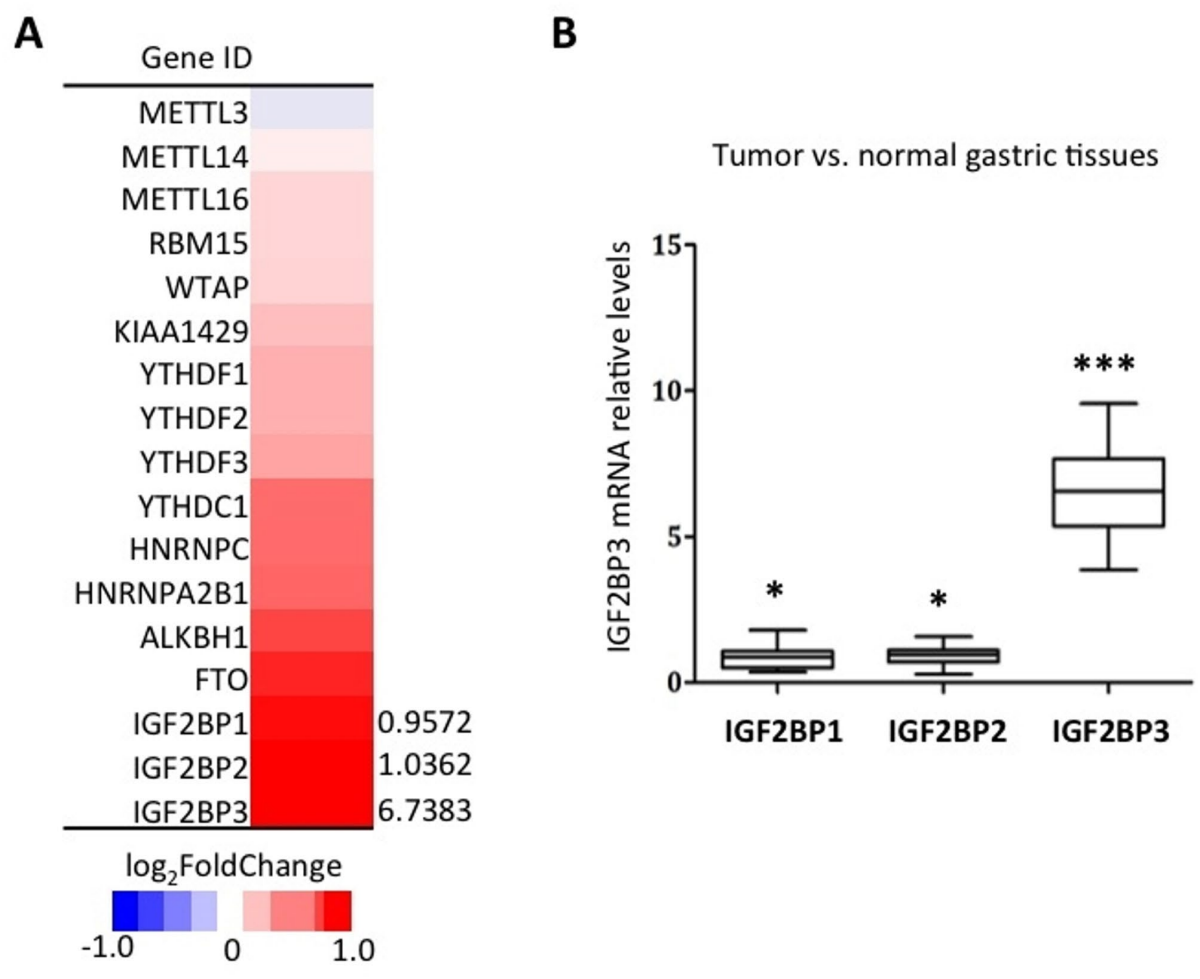
Comparative analysis of m6A-related regulator expression in GC and normal gastric tissues. (**A**) heatmap visualization of significantly upregulated m6A-related regulators in GC tissues. (**B**) qPCR validation of *IGF2BP* family overexpression. *, *p* < 0.05; ***, *p* < 0.0001

In general, m6A modification is regulated by three groups of enzymes: “writers” (METTL3/14/16), “readers” (YTHDF1/2/3, IGF2BP1/2/3), and “erasers” (FTO/ALKBH5). Comparative analysis of regulator mRNA levels between tumor and adjacent normal tissues was conducted to assess their association with the dysregulation of transcriptome and/or methylome in GC tissues. Transcriptomic analysis revealed widespread activation of these regulators in GC tissues, with METTL3 being the sole exception (Fig. 7A). Notably, the IGF2BP family members (*IGF2BP1/2/3*) exhibited the most pronounced upregulation among all regulators examined.

We then validated the IGF2BP expression patterns in 58 matched GC and adjacent normal tissue pairs using quantitative real-time polymerase chain reaction (qPCR). The expression of *IGF2BP3* was significantly elevated in GC tissues (fold change > 106, *p* < 0.0001), while *IGF2BP1* and *IGF2BP2* showed more modest but statistically significant increases (*p* < 0.05) (Fig. 7B).

Patients with GC were divided into high- and low-*IGF2BP3* expression groups. We then analyzed the association between *IGF2BP3* expression and the clinicopathological characteristics of patients with GC. As summarized in Table 2, the expression level of *IGF2BP3* was significantly associated with multiple GC characteristics, including lymph node involvement (*p* = 0.016) and tumor-node-metastasis (TNM) staging (*p* = 0.028).

**Table 2:** Association between *IFG2BP3* expression and characteristics of patients with GC

Clinicopathological characteristics	IGF2BP3 levels		Chi-squared value	*p*-value
	High (*n* = 30)	Low (*n* = 28)		
Age (years)				
≥60 <60	17 13	15 13	0.0561	0.8128
Sex				
Male Female	18 12	17 11	0.0031	0.9557
Tumor location				
Cardia Non-cardia	11 19	9 19	0.1312	0.7172
Histological differentiation				
Moderate Poor	8 22	7 21	0.0291	0.8848
TNM stage				
I + II III + IV	5 25	12 16	4.7950	0.0285*
Lymph node involvement				
No Yes	6 24	14 14	5.7690	0.0163*
Depth of invasion				
T1–T2 T3–T4	8 22	13 15	2.4490	0.1176

TNM, tumor-node-metastasis; *, *p* < 0.05; *n*, number

## Discussion

N6-methyladenosine (m6A), the most abundant internal mRNA modification in eukaryotes [20], serves as a critical regulator of RNA metabolism through its involvement in mRNA processing, stability control, and translational regulation [21, 22]. Studies have shown that m6A gene modification plays a crucial role in tumorigenesis by modulating the expression of critical genes associated with cancer phenotypes [23–25]. While previous research has primarily focused on alterations in m6A methylation regulators in GC, the genomic genes specifically modified with m6A in GC and their functions remain poorly characterized.

In this study, we performed MeRIP-seq to determine the m6A modification landscape in paired GC and adjacent normal tissues. Consistent with established patterns in mammalian systems [26, 27], we observed predominant m6A enrichment near stop codons and 3’UTRs. We found that m6A peaks in both GC and normal tissues were spread along the transcript and mostly enriched in the CDA close to the stop codon - regions known to influence RNA stability, transport, and translation. Notably, our comparative analysis revealed significantly higher m6A methylation levels in CDS regions of GC tissues compared to normal controls. These findings suggest that CDS-specific m6A hypermethylation, potentially induced by genetic alterations or microenvironmental factors, may represent a novel mechanism contributing to gastric carcinogenesis. However, further studies are required to validate this hypothesis.

A recent study investigated the m6A methylation profiles of three paired GC tissues and found that genes with differential m6A modifications in GC were enriched in the transcriptional dysregulation and digestion/absorption pathways [28]. In contrast, our comprehensive profiling demonstrated widespread significantly downregulation of differential m6A peaks, indicating global hypomethylation as a characteristic feature of GC tissues. This global hypomethylation pattern aligns with the proposed model by Zhang et al., wherein the loss of m6A modifications may preferentially promote GC progression through oncogenic signaling pathway activation [29]. Functional enrichment analysis of m6A-hypomethylated genes in our study identified significant involvement in metabolic pathways, a finding with particular relevance given that metabolic reprogramming is a hallmark of cancer development and is highly intertwined with the epigenetic inheritance of cancer cells [30, 31]. Beyond identifying metabolic pathways as key targets of m6A hypomethylation in GC, our findings raise important questions about the mechanistic connections between RNA methylation and cancer metabolism. Several potential mechanisms may explain this association: (i) m6A-mediated regulation of the critical key enzymes of the Warburg effect or aerobic glycolysis: Wang et al.‘s study demonstrated that METTL3 enhances the stability of one of the critical enzymes for the Warburg effect, HK2, through YTHDF1-mediated m6A modification, thereby promoting the Warburg effect in cervical cancer [32]. Our observation of hypomethylation in metabolic genes suggests a similar mechanism may operate in GC. (ii) Epitranscriptomic control of metabolic master regulators: The m6A machinery has been shown to target transcription factors and signaling nodes that orchestrate metabolic reprogramming. A study by Liu et al. [33] revealed that m6A modification of HIF1α mRNA regulates its translation under hypoxia via the ALKBH5-HDAC4-HIF1α axis, directly linking RNA methylation to metabolic adaptation. Our data showing transcriptional misregulation (Fig. 5B) suggest this could represent another layer of metabolic control in GC. (iii) Cross-talk between m6A and mitochondrial metabolism: Emerging evidence indicates specialized roles for m6A in mitochondrial-encoded transcripts. The significant hypomethylation we observed in genes related to primary metabolic processes (Fig. 5A) may reflect a broader disruption of mitochondrial RNA metabolism in GC, consistent with recent reports demonstrating that METTL14-mediated m6A loss reduces mitochondrial respiratory chain complex activity and supercomplex assembly, and that the METTL3-YTHDF1 axis regulates oxidative phosphorylation dysfunction in metabolic disorders [34, 35]. Several studies have identified significant metabolic alterations within the tumor microenvironment and gastric secretions [36–42]. Hypomethylation of genes in metabolic pathways may provide novel insights into GC pathogenesis at the epigenetic level.

Functional enrichment analysis demonstrated that transcripts exhibiting m6A hypomethylation were significantly enriched in transcriptional dysregulation pathways and were involved in transcriptional regulation and DNA-binding functions. The hypomethylation of transcriptional regulators may alter signal recognition motifs and compromise the stability of oncogenic transcripts. Through comparative transcriptomic analysis of GC and adjacent normal tissues, we identified m6A-modified genes with differential expression patterns. Notably, while the majority of transcripts were downregulated in GC tissues (721 of 1,157), only five specific genes (*TOM1L2*, *ST5*, *STEAP3*, *LDOC1*, and *GLUL*) exhibited concurrent mRNA-level reduction and m6A hypomethylation. This limited overlap may be attributed to several factors: (i) Temporal dynamics of m6A regulation: m6A modifications predominantly influence post-transcriptional processes such as mRNA stability, splicing, and translation efficiency rather than transcriptional output. Wang et al. [43] have demonstrated that m6A modifications can dynamically regulate mRNA decay without altering steady-state transcript levels, explaining why many genes show m6A changes without corresponding expression shifts. (ii) Context-dependent effects of m6A: The functional consequences of m6A modifications are highly context-dependent, influenced by the presence of specific “reader” proteins (e.g., YTHDF1/2/3 or IGF2BP family members). As highlighted by Huang et al. [44], IGF2BPs stabilize target mRNAs in an m6A-dependent manner, whereas YTHDF2 promotes decay. Thus, even with reduced m6A levels, the net effect on mRNA abundance may vary based on the dominant ‘reader’ machinery in GC cells. (iii) Compensatory mechanisms: Cancer cells often activate compensatory pathways to buffer against transcriptomic perturbations. For example, METTL3 knockdown in glioblastoma reduces m6A levels but upregulates alternative splicing factors to maintain transcript homeostasis [45]. Such redundancy could decouple m6A changes from observable expression differences. A distinctly different pattern was found in the KEGG analysis of DEGs in tumor tissues. DEGs were mainly enriched in pathways related to protein digestion and absorption, extracellular matrix-receptor interactions, gastric acid secretion, and cell cycle regulation. Given the post-transcriptional nature of m6A-mediated gene regulation, DEGs in GC may not directly reflect alterations in their m6A methylation levels. Transcriptome alterations in GC tissues may be a downstream effect of dysregulation of m6A modification.

Recent studies have established the critical involvement of m6A-modified genes in tumor initiation and malignant progression [5, 6]. The seminal work by Sun et al. [46] systematically characterized the role of m6A-related genes in GC. Their comprehensive analysis of 15 core m6A-related genes (*WTAP*, *KIAA1429*, *RBM15/15B*, *METTL3/14/16*, *HNRNPA2B1*, *HNRNPC*, *YTHDC1/F1/F2/F3*, *FTO*, and *ALKBH5*) revealed significant dysregulation in GC tissues compared to normal gastric tissues. They identified *FTO* upregulation as a potential independent prognostic factor for recurrence-free survival in patients with GC. *IGF2BPs* are newly recognized m6A-related genes that function as “readers” to enhance mRNA stability and translation [44]. Our comparative analysis revealed significant overexpression of *IGF2BPs* in GC tissues compared to adjacent normal tissues, with *IGF2BP3* showing the most pronounced upregulation (fold change > 106). These findings suggest *IGF2BP3* as a potential diagnostic and prognostic biomarker for GC.

We further identified significant positive correlations between elevated *IGF2BP3* expression and aggressive clinicopathological parameters in GC, particularly lymph node involvement and TNM staging. These findings implicate *IGF2BP3* in GC progression, consistent with its established oncogenic role across multiple malignancies. For instance, *IGF2BP3* overexpression has been documented to promote tumor invasiveness and predict adverse outcomes in breast carcinoma and lung adenocarcinoma [47, 48]. In recent studies, *IGF2BP3* was also confirmed as a potential oncogene that promotes GC progression [19, 49]. While current mechanistic understanding has primarily focused on microRNA-*IGF2BP3* interactions, the comprehensive molecular pathways remain incompletely investigated. Our study provides evidence supporting the role of *IGF2BP3* in GC progression through m6A methylation dysregulation.

Our results showed a genome-wide reduction in m6A modifications in GC tissues compared with adjacent normal tissues. These hypomethylated transcripts were functionally enriched in metabolic pathways and transcriptional dysregulation in cancer. Notably, we observed widespread activation of m6A-related genes in GC, with *IGF2BP3* emerging as the most significantly upregulated effector. Clinicopathological correlation analysis established *IGF2BP3* overexpression as strongly associated with multiple characteristics of patients with GC, including lymph node involvement and TNM staging.

## Conclusion

We demonstrate that there was a transcriptome-wide reduction of m6A modifications in GC tissues, highlighting the role of m6A methylation dysregulation in driving GC progression. Our findings also identify *IGF2BP3* as a promising therapeutic target and prognostic biomarker in GC.

## Supplementary Information

Below is the link to the electronic supplementary material.

Supplementary Material 1

## Data Availability

No datasets were generated or analysed during the current study.

## Abbreviations

m6A: N6-methyladenosine
MeRIP: Methylated RNA immunoprecipitation
RNA-seq: RNA sequencing
CDS: Coding sequence
ATP: Adenosine triphosphate
DEGs: Differentially expressed genes
ECM: Extracellular matrix
qPCR: Quantitative real-time polymerase chain reaction
TNM: Tumor-node-metastasis
UTRs: Untranslated regions of mRNAs
