# Supplementary material for: Clinical and prognostic significance of m6A hypomethylation and IGF2BP3 overexpression in gastric cancer: an integrated epigenomic-transcriptomic analysis
Source: Hum Genomics. 2025 Aug 22;19:95. doi: 10.1186/s40246-025-00802-0 (PMC12374356; doi:10.1186/s40246-025-00802-0)
Supplement: Supplementary file 1 — Supplementary Material 1 [file 40246_2025_802_MOESM1_ESM.docx]

| **Gene Name** | **Forward Primer (5'→3')** | **Reverse Primer (5'→3')** |
| --- | --- | --- |
| *ATF6* | GCCTTTATTGCTTCCAGCAG | TGAGACAGCAAAACCGTCTG |
| *MRPL28* | TCTGAACCCTGAAAGGCTCG | GCCTATAGTGCACGGGAGTG |
| *ZNF329* | AATCGCCCGGAACATGGC | CCAGCCATCTCTGAAGTCCC |
| *RAB3D* | GCCATCTTCCAGCCCTGTTGTGC | CTCAGTTATGGAGCAAGGCTACCG |
| *CPPED1* | CAGCGACACACATTGCTCT | GCAGGGGTGCGTACAGAAT |
| *SHANK3* | GGGATCACCGACGGAATATGG3 | TGTCTGCCCCATAGAACAGC |
| *ZNHIT2* | GGATAATGCCCCGGGTAGTG | CGTAGGCGAACAGCACATTG |
| *POLR3H* | CCTCTGGCCTCAGTTTCCTC | CACCATTTCCACCAGGACGA |
| *PPP1R37* | AACGAGATCAAGACAGGCGG | TCTGCGTCTCGATGAAGCTC |
| *RAMACL* | TGAGTCAGCTCCACACTTGAG | TTGTGTGCAATTGTCCAGGC |
